# Psychopathology and health-related quality of life as patient-reported treatment outcomes: evaluation of concordance between the Brief Symptom Inventory (BSI) and the Short Form-36 (SF-36) in psychiatric outpatients

**DOI:** 10.1007/s11136-021-03019-5

**Published:** 2021-11-02

**Authors:** Edwin de Beurs, Ingrid Carlier, Albert van Hemert

**Affiliations:** 1 Department of Clinical Psychology, Leiden University, Leiden, Netherlands; 2 Arkin Mental Health Institute, Amsterdam, Netherlands; 3 Department of Psychiatry, Leiden University Medical Centre, Leiden, Netherlands

**Keywords:** BSI, SF-36, Patient-reported outcome measures (PRO), Routine outcome monitoring (ROM), Treatment evaluation, Common mental disorders

## Abstract

**Purpose:**

Treatment outcome for common psychiatric disorders, such as mood and anxiety disorders, is usually assessed by self-report measures regarding psychopathology [e.g., via Brief Symptom Inventory (BSI)]. However, health-related quality of life [as measured by the 36-item Short-Form Health Survey (SF-36)] may be a useful supplementary outcome domain for routine outcome monitoring (ROM). To date, the assessment of both outcomes has become fairly commonplace with severe mental illness, but this is not yet the case for common psychiatric disorders. The present study examined among outpatients with common psychiatric disorders whether aggregate assessments of change across treatment regarding psychopathology and health-related quality of life yield similar results and effect sizes.

**Methods:**

We compared treatment outcome on the BSI and the SF-36 in a sample of 13,423 outpatients. The concordance of both instruments was assessed at various time points during treatment.

**Results:**

Scores on both instruments were associated, but not so strongly to suggest they measure the same underlying construct. The SF-36 scales presented a varied picture of treatment outcome: understandably, patients changed more on the mental component scales than on physical component scales. Outcome according to the BSI was quite similar to outcome according to scales of the SF-36 that showed the largest change.

**Conclusions:**

Although (mental health) scores on both instruments are associated, adding the SF-36 in addition to the BSI in treatment evaluation research produces valuable information as the SF-36 measures a broader concept and contains physical/functional component scales, resulting in a more complete clinical picture of individual patients.

## Introduction

Measurement-based care (MBC) uses patient-reported rating scales in conjunction with evidence-based clinical practice guidelines to provide an objective assessment of patient progress over time to guide a more precise plan of care [1], prevent treatment failure [2], and collect data for quality management [3]. Through the use of patient-reported outcome, PROs [4], the patient’s voice is being heard, quantified, and compared to normative data in a large variety of domains [5]. This is in line with international trends, where more emphasis is put on the value of health care in terms of outcome [6] and patients are granted a more prominent role [7].

Current health definitions involve at least three domains: physical, mental, and social health that should be prioritized in delivering health care [8, 9]. Ideally, health outcomes should include all three domains of health in a full cycle of care [10]. In somatic care (e.g., oncology), the use of MBC has become largely routine, and usually several health domains are measured (e.g., physical symptoms, functioning, anxiety, depression) [1]. In psychiatric care, on the other hand, MBC is less standard practice, due to several barriers, such as lack of agreement on key outcome domains and lack of empirical data on outcome measures [3]. Measurement often mainly focuses on mental health without including other measurement domains, such as functioning or wellbeing [11, 12].

In mental health care research, outcome is commonly assessed by comparing the severity of psychopathology before and after treatment with generic or disorder-specific instruments. In this context, a widely used instrument is the Brief Symptom Inventory, BSI [13, 14], which provides with its total score (the Global Severity index) information regarding the severity of general psychopathology as well as on specific symptoms, such as depression and anxiety. In assessing the severity of psychopathology, these instruments measure signs and symptoms of the disorder (e.g., those listed in the prevailing taxonomy of mental disorders, the DSM-5).

However, operationalization of outcome in mental health care by signs and symptoms has been criticized as too narrow and too much focused on deficits [10, 15]. Health is more than the absence of signs and symptoms. Patients’ view on their health-related quality of life (HRQOL) offers a broader conceptualization and may yield a useful additional indicator of treatment outcome. HRQOL is defined as the quality of life relative to one’s health or disease status, and it is commonly conceived as dynamic, subjective, and multidimensional [16]. This shift in emphasis is also reflected in the emergence of the recovery movement in psychiatry, with its distinction between clinical and personal recovery [17], and positive psychology [18, 19] as well as positive psychiatry movements [20].

An instrument for the assessment of HRQOL is the Short Form-36, SF-36 [21, 22], widely used in health care and mental health care. The existing literature on HRQOL in mental health care research is predominantly concerned with severe mental disorders, such as psychotic disorders [23–25], for which the measurement of HRQOL is seen as a necessary addition to other outcome domains such as psychopathology. For common mental disorders, such as mood- and anxiety disorders, the value of adding HRQOL to the assessment of treatment outcome is less well investigated. Assessment of HRQOL in mood disorders has been recommended [26], but little research comparing measures head-to-head has been done [27]. In a meta-analyses for anxiety disorders, Olatunji et al. [28] reported that Post-Traumatic Stress Disorder (PTSD) in particular was associated with decreased HRQOL. This finding was confirmed in a recent study with PTSD patients [29], demonstrating a strong association between the change in depression symptoms and change in HRQOL, which could be expected as depression symptoms are incorporated in HRQOL measures.

Thus, there is a strong plea for a broader assessment of the benefits of mental health care than mere symptom relief [30]. Adding an outcome domain to signs and symptoms is especially valuable when a decrease in signs and symptoms correlates only moderately with increased health-related quality of life. This may be the case when change over time on both constructs does not occur in synchrony. Many patients fist show improvements in symptomatology, to be followed later on by an increase in health-related quality of life. However, the precise association between symptomatology and quality of life in MHC is still poorly understood [31].

The present study used routine outcome monitoring (ROM) data of outpatients with common mental disorders to investigate and compare both outcome domains. Longitudinal data of the BSI and the SF-36 were compared, their correlation assessed, and—more importantly—the concordance between a decrease in score on the BSI with an increase in score on the SF-36 over time was established. We investigated whether the overall magnitude and the pace of change over time was similar in both domains. After all, a common hypothesis is that therapeutic change is first manifest on symptoms (in this case measured by the BSI), to be followed later by improved functioning or HRQOL [32, 33]. We analyzed this issue using a subset of the sample with four repeated assessment per patient, which enabled us to test this hypothesis regarding the dyssynchrony of response on the two outcome domains of psychopathology and HRQOL.

In sum, the main objective of the study was to investigate and compare the responsiveness of two outcome measures: a symptom checklist (BSI) and a HRQOL measure (SF-36). An asynchronous response pattern on both constructs was hypothesized: Symptoms decrease first, followed later on by an increase in quality of life.

## Methods

### Procedure

The collection of data is described in detail by de Beurs et al. [34]. Here, we provide a brief description. For this study, we used data from the Dutch Center of Routine Outcome Monitoring (COROM) from 2003 to 2013. ROM data have been collected at the Leiden University Medical Centre (LUMC, Department of Psychiatry) and at the Mental Health Care Provider GGZ Rivierduinen (the main service provider in the northern half of the South-Holland province, an area with 1.1 million inhabitants). Outpatients with mood-, anxiety-, and/or other psychological problems were referred for treatment by their general practitioner. All patients were informed that ROM was part of the intake process. They were interviewed by a research nurse about their psychiatric symptoms with a semi-structured diagnostic interview (MINI-Plus [35, 36]). Next, patients completed a set of generic and disorder-specific questionnaires. The selection of disorder-specific questionnaires was based on the outcome of the MINI-Plus [35]. During treatment, they were assessed every three to four months with a comprehensive battery of self-report instruments and rating scales. Per sampling round, the number of patients decreased by 50%, partly due to the completion of treatment, partly because of no-show (after repeatedly being contacted by mail, email, and phone) of the patient at the test session. About half the patients discontinued ROM, but continued treatment. For the other half, the last available assessments coincided with ending the treatment and can be considered as the posttest assessment [34]. The mean measurement interval after the pretest was a little over 6 months, from the 2nd to 3rd and from the 3rd to the 4th measurement a little short of 6 months (see Table 2).

### Study population

Initially, data of 13,811 psychiatric outpatients were available; 5826 were retested at least once with the BSI and the SF-36 and we used this sample for analyses regarding responsiveness (628 cases were excluded, based on negative retest intervals or a retest interval < 2 weeks as these were considered administrative errors; also, cases where the assessment on the BSI and the SF-36 were more than a week apart were excluded). Table 1 presents demographic and clinical data on the sample with pre- and retest data and the sample with four assessments. Most patient suffered from a Major Depressive Disorder or an anxiety disorder (singular or comorbid). A substantial number of patients (about 20 to 25%) did not meet criteria for a current DSM-IV disorder according to the MINI-Plus. Table 2 presents data regarding loss to follow-up; for 1463 patients at least four assessments were available. The pretest scores of the sample with a single retest and the subsample with four assessments were similar**.**

**Table 1:** Demographic and pretest clinical characteristics of the sample with at least two assessments (*n* = 5826) and the sample with four assessments (*n* = 1463) and posttest scores for the latter sample

Diagnosis (DSM-IV):	Twice assessed		Four times assessed			
	Pretest		Pretest		Posttest	
	*N*	%	*N*	%		
Gender (female)	3674	63.1	929	63.5		
Age (*M*, SD)	39.1	12.7	39.1	13.0		
Diagnosis						
Singular mood disorder	1717	29.5	467	31.9		
Singular anxiety disorder	1295	22.9	297	20.3		
Comorbid mood/anxiety	1164	20.0	353	24.1		
Singular other disorder^a^	43	0.7	13	0.9		
Comorbid mood/other^a^	55	0.9	18	1.2		
Comorbid anxiety/other^a^	30	0.5	9	0.6		
Comorbid mood/anxiety/other^a^	64	1.1	20	1.3		
No DSM-IV disorder	1457	25.0	288	19.7		

	*M*	*SD*	*M*	*SD*	*M*	*M*
BSI: TOT	1.23	0.70	1.32	0.69	0.83	0.65
DEP	1.56	1.00	1.69	0.99	0.96	0.89
ANX	1.39	0.94	1.49	0.95	0.90	0.81
SF-36: PCS	49.1	10.7	48.6	10.7	49.3	9.9
MCS	28.0	11.3	26.5	10.9	36.8	12.5
PF	76.4	23.4	74.7	23.2	79.8	22.2
RP	40.3	41.0	37.4	40.3	53.0	41.9
BP	68.3	27.1	67.4	26.8	72.6	25.1
GH	53.1	21.0	51.2	20.2	57.9	21.3
VT	36.1	18.4	33.6	17.7	47.4	20.7
SF	47.4	26.6	44.3	26.0	63.7	26.6
RE	30.7	37.5	27.3	36.0	50.9	42.2
MH	44.1	18.8	41.5	18.3	57.7	19.9

*BSI: TOT* total score, *DEP* Depression, *ANX* Anxiety, *SF-36: PCS* Physical Component Score, *MCS* Mental Component Score, *PF* Physical Functioning, *RP* Role limitations Physical, *BP* Bodily Pain, *GH* General Health perceptions *VT* Vitality, *SF* Social Functioning, *RE* Role limitations Emotional, *MH* Mental Health

^a^Somatoform disorder, eating disorder, addictive disorder, adult attention-deficit/hyperactivity disorder

**Table 2:** Number of patients at each assessment and the length of various time intervals

	1st	2nd	3rd	4th assessment
Entire dataset	13,811	6579	3392	1723
Both BSI and SF-36 present	13,432	6454	3329	1694
With outliers removed^a^	12,806	5826	2946	1463
Reassessment after *M* (SD) days		197 (142)	172 (117)	170 (110)

^a^628 Cases (9.7%) were removed, due to negative, extremely short or extremely long time intervals

Patients were treated according to evidence-based guidelines with pharmacological and/or psychological treatments (mainly Cognitive Behavior Therapy [37]). The Medical Ethical Committee of the LUMC approved the general study protocol regarding ROM, in which ROM is considered integral to the treatment process (no written informed consent is institutionally required for the analysis of anonymized data). A comprehensive protocol (Psychiatric Academic Registration Leiden database) was used, which safeguarded the anonymity of participants and ensured proper handling of the data. All participants gave permission for the anonymized use of their data for scientific study.

### Instruments

#### Mini-International Neuropsychiatric Interview Plus

The MINI-Plus [35] is a fully structured diagnostic interview that assesses DSM-IV criteria for the main psychiatric disorders (current/lifetime) such as mood-, anxiety-, somatoform-, substance use-, psychotic-, eating-, conduct, attention-deficit/hyperactivity, adjustment-, and anti-social personality disorders. It is organized in 26 modules: affirmative answers to screening questions are explored by establishing the presence of additional diagnostic criteria. Excellent inter-rater and test–retest reliabilities of the MINI have been established [35].

#### Short Form-36

The SF-36V1 [21, 22], also known as the RAND-36 or MOS-36, is a widely used instrument for measuring HRQOL or functional health status. The 36 items refer to the last 4 weeks, except for the scale Physical Functioning (PF) (“at this moment”) and General Health Perceptions (GHP) (“in general”). The items have measurement scales of different lengths (2-, 3-, 5-, and 6-point scales), which are converted to a 0–100 scale before they are averaged into scale scores. Items are allocated to eight subscales: *Physical Functioning (PF), Role limitations due to Physical problems (RP), Bodily Pain (BP), General Health perceptions (GH), Vitality (VT), Social functioning (SF), Role limitations due to Emotional problems (RE), and general Mental Health (MH).* In addition, scores for two orthogonal health components can be calculated from the SF-36 scales: the physical and the mental component, respectively, PCS (primarily reflecting PF, RP, BP, and GH) and MCS (primarily reflecting VT, SF, RE, and MH). The component scores are based on Principal Component Analysis of SF-36 scale scores derived from the American general population as recommended by Ware et al. [38]. Together, they explain 80 to 85% of the variance in scale scores and the components have proved reliable and valid. Component scores are created by multiplying standardized scale scores, based on Dutch norms [22], with the component score coefficients of the two-component solution [39]. A higher score means better health.

#### Brief Symptom Inventory

The BSI [13, 14] is one of the most frequently used general symptom measures in mental health care regarding psychopathology. It consists of 53 items (a selection of the best performing items of the SCL-90, the precursor of the BSI), each describing a “problem” (complaint or symptom). The respondent is asked to indicate “how he/she has been affected by this problem, the past week including today” on a 5-point Likert scale, ranging from 0 = none to 4 = very much. The BSI includes nine scales, each with four to six items: somatic symptoms, cognitive symptoms, interpersonal sensitivity, depression, anxiety, hostility, phobic symptoms, paranoia, and psychoticism. Furthermore, a total score can be calculated representing severity of general psychopathology. For the present study, the total score is used (*BSI–TOT*) as well as the Depression (*BSI-DEP*) and Anxiety (*BSI-ANX*) subscales. A higher score means more psychopathology. The BSI and the SF-36 are both patient-reported outcome (PRO) measures.

### Statistical analyses

To get a similar direction in scores on the BSI and the SF-36, the SF-36 scales were reversed. In addition, scores on the BSI and the SF-36 scales were transformed to the same metric by subtracting the pretest mean score and dividing it by the pretest standard deviation (standard or *Z*-scores). Thus, for the entire sample, the initial score is *M* = 0 (SD = 1), and difference scores from pretest to subsequent assessments are of the same size as the within-group effect size (ES) estimator: ES = (*M*_pretest_ − *M*_posttest_)/SD_pretest_) [40]. For ES, 0.20 indicates a small effect, 0.50 a medium effect, and 0.80 a large effect [41]. For the analysis of the longitudinal data, a subgroup with at least four assessments was selected, as this yielded the most optimal balance between the number of retests and loss to follow-up. Furthermore, we established the ES of the first assessment interval (ES_1–2_) and for the interval between the first and the last available complete assessment of each patient in the entire study group (ES_MAX_).

To assess construct validity of the BSI and the SF-36, we determined the correlation between scores at pretest and at the final assessment; to compare responsiveness we determined correlations between change scores (pre-to-retest difference) from the first to the second assessment and from the first to the final assessment [42]. To detect dissimilarity and asynchrony of response on the BSI and the SF-36, we compared the course of scores on both instruments over time with multivariate analysis of variance for repeated measurements. In this analysis, a significant interaction effect between time and instrument indicates a difference between instruments in change over time. For the instrument effect, after the omnibus test a “simple” contrast was tested, which compared the BSI total score with each SF-36 component and scale score. The repeated contrast for time compares the first with the second assessment, the second with the third, and so on. With four time points, it can be determined whether there is a temporal difference in change according to the two outcome measures (e.g., first a change in psychopathological symptoms (BSI), followed by a change in health-related quality of life (SF-36) in the ensuing time interval). The large number of observations per analysis (generally *n* > 1500) provides abundant statistical power to find differences. Consequently, also small differences will be statistically significant. Therefore, it is more important to look at the proportion of variance accounted for by each statistically significant effect [43]. For the between-instrument effect, the time effect, and the interaction effect, we present partial *η*^2^ as provided by SPSS. For *η*^2^, 0.01 indicates a small effect; 0.06 a medium effect; 0.14 a large effect [41].

## Results

### Concordance of the BSI–TOT with SF-36 scales

A first step to detect dissimilarity and asynchrony of response on the BSI and the SF-36 was to investigate the correlation between both measures. Table 3 shows Pearson correlation coefficients for the association of the total score on the BSI and the SF-36 scale scores. The correlation coefficients of the BSI with the component and scale scores were generally between *r* = .40 and .70, indicating that the BSI and the SF-36 measure different but related concepts. In accordance with its purported measurement aim, the SF-36 general Mental Health scale (MH) correlates the strongest with the BSI total score; second highest is the Mental Component Score (MCS). This applies to both the baseline data and the last available assessment. Correlation between change scores for the first measurement interval and the maximum measurement interval are lower, but change scores are still substantially associated. Again, the highest correlations are found between the BSI and the MCS and MH scales. Furthermore, results appear to stabilize over time, as correlations between component and scale scores are systematically lower at the first assessment versus the last available assessment, and the fist interval and the last interval.

**Table 3:** Correlation (product moment correlation coefficients) between BSI total score and the SF-36 component and scale scores at the first and the last available (*n*th) assessment and between difference scores

BSI total score	PCS	MCS	PF	RP	BP	GH	VT	SF	RE	MH
1st Assessment (*n* = 13,432)	.25	.66	.35	.33	.39	.43	55	.57	.47	.75
*n*th Assessment (*n* = 5826)	.36	.76	.44	.51	.48	.57	.66	.69	.60	.82
Difference score 1st interval	.14	.64	.27	.30	.26	.39	.54	.52	.40	.70
Difference score *n*th interval	.16	.66	.30	.34	.28	.42	.57	.57	.44	.72

All correlations are two-sided significant at *p* <  .001

*PCS* Physical Component Score, *MCS* Mental Component Score, *PF* Physical Functioning, *RP* Role limitations Physical, *BP* Bodily Pain, *GH* General Health perceptions, *VT* Vitality, *SF* Social Functioning, *RE* Role limitations Emotional, *MH* Mental Health

### Gradient over time

**Fig. 1:**
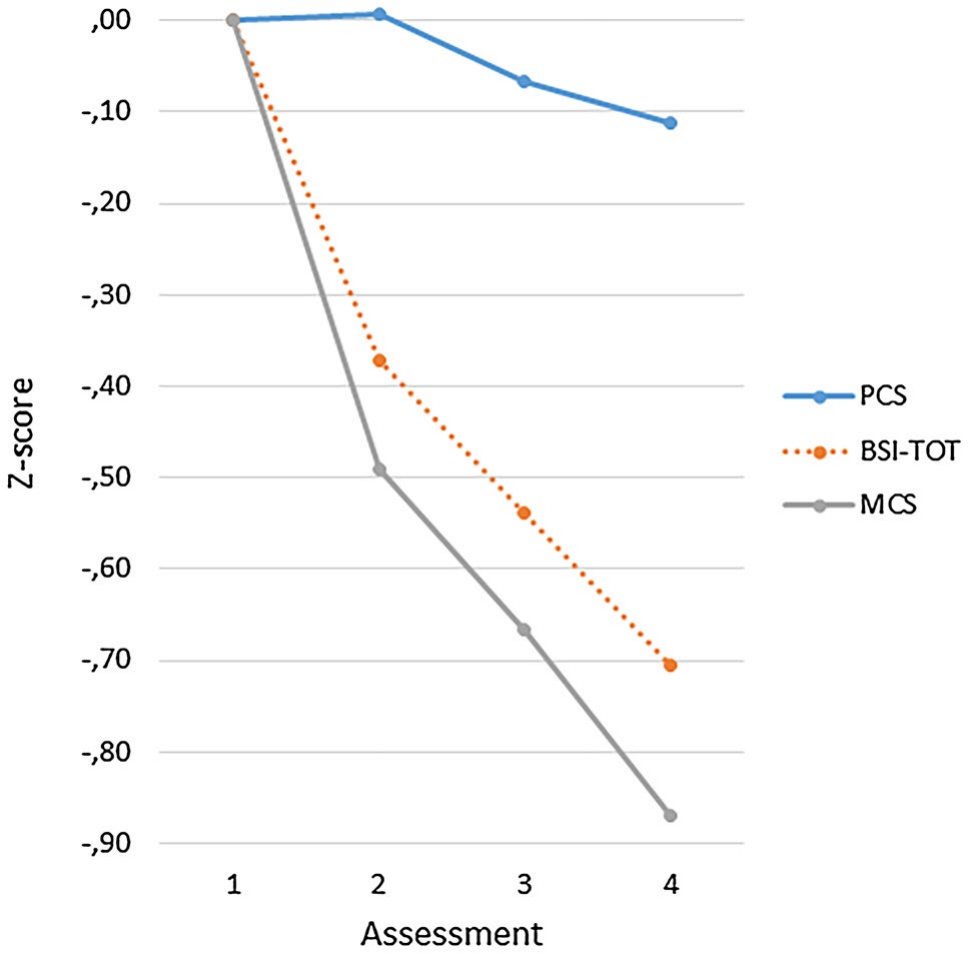
Course over time of standardized *Z*-scores on the BSI total score and SF-36 Physical and Mental components for the group with four assessments (*n* = 1453)

The data of the group with four assessments (*n* = 1463; mean interval of first to fourth measurement *M*_1–4_ = 471.9 days, SD = 184.1) were analyzed with repeated measures analysis to compare the course over time of the BSI total score with the two SF-36 component scores for physical and mental health (PCS and MCS). This revealed main effects of time ([*F*(3, 1460) = 307.46; *p* < .001, *η*^2^ = .387), instrument [*F*(2, 1460) = 84.38; *p* < .001, *η*^2^ = .104], and a significant interaction effect for instrument-by-time [*F*(6, 1457) = 74.86; *p* < .001, *η*^2^ = .236]). On each consecutive assessment, scores diminished on the BSI and increased on the SF-36, indicating that, on average, patients improved over time. Table 4 presents the values of partial *η*^2^ for main effects and interaction effect of the time-by-instrument factorial model. The second column presents partial *η*^2^ for the overall effect, later columns present partial *η*^2^ for repeated contrasts. Contrasts comparing the consecutive assessments (repeated contrast) show that the largest change occurs in the first assessment interval. The instrument effect was predominantly due to the BSI–PCS contrast (less change over time for the PCS). The significant time-by-instrument interaction was predominantly due to the difference in change according to the BSI and the PCS in the first interval. The statistical significance of the effects indicates that the BSI differed from the PCS for all intervals, but it differed only in the first interval significantly from the MCS (with a larger change on the MCS). Analyses of standardized mean differences (partial *η*^2^) corroborate this finding. Figure 1 clarifies these findings graphically as it shows the course of scores over time for patients with at least four assessments. The size of *η*^2^ for the time contrast effect in Table 4 indicates that the lines in Fig. 1 deviate significantly from a horizontal course (the greatest decrease in score is attained in the starting phase); the statistical significance and size of *η*^2^ for the interaction effect of time * instrument indicates that the lines do not run parallel. The difference between the BSI and the PCS was substantial (partial *η*^2^ = .125 for the first interval). In the first interval, there was a larger change on the MCS compared to the BSI, but no difference between both scores in the last two time intervals. Thus, our hypothesis of an asynchronous response between the BSI and the SF-36, with scores on the BSI decreasing first, is not supported by the findings.

**Table 4:** Overview of effect sizes (partial *η*^2^) for main and interaction effects of the analysis of variance for repeated measures using contrasts to compare BSI–TOT with the SF-36 component scores over four consecutive assessments (repeated contrast)

	Overall		Repeated contrast		
Time effect	**.224**		From 1–2	From 2–3	From 3–4
			**.187**	.055	.057
		Contrast			
Instrument effect	.084	BSI–TOT vs. SF-36–MCS	.033		
		BSI–TOT vs. SF-36–PCS	.078		
			Repeated contrast		
Time * instrument	.082	Contrast	From 1–2	From 2–3	From 3–4
		BSI–TOT vs. SF-36–MCS	.018	.000*	.002*
		BSI–TOT vs. SF-36–PCS	.125	.009	.019

*BSI–TOT* Brief Symptom Inventory Total Score, *SF-36–PCS* SF-36 Physical Component Score, *SF-36–MCS* SF-36 Mental Component Score

*Small effects and interaction effects were not statistically significant (*p* > .10); partial *η*^2^ = 0.01 indicates a small effect, *η*^2^ = 0.06 indicates a medium effect, and *η*^2^ = 0.14 is a large effect (in bold typeface)

**Fig. 2:**
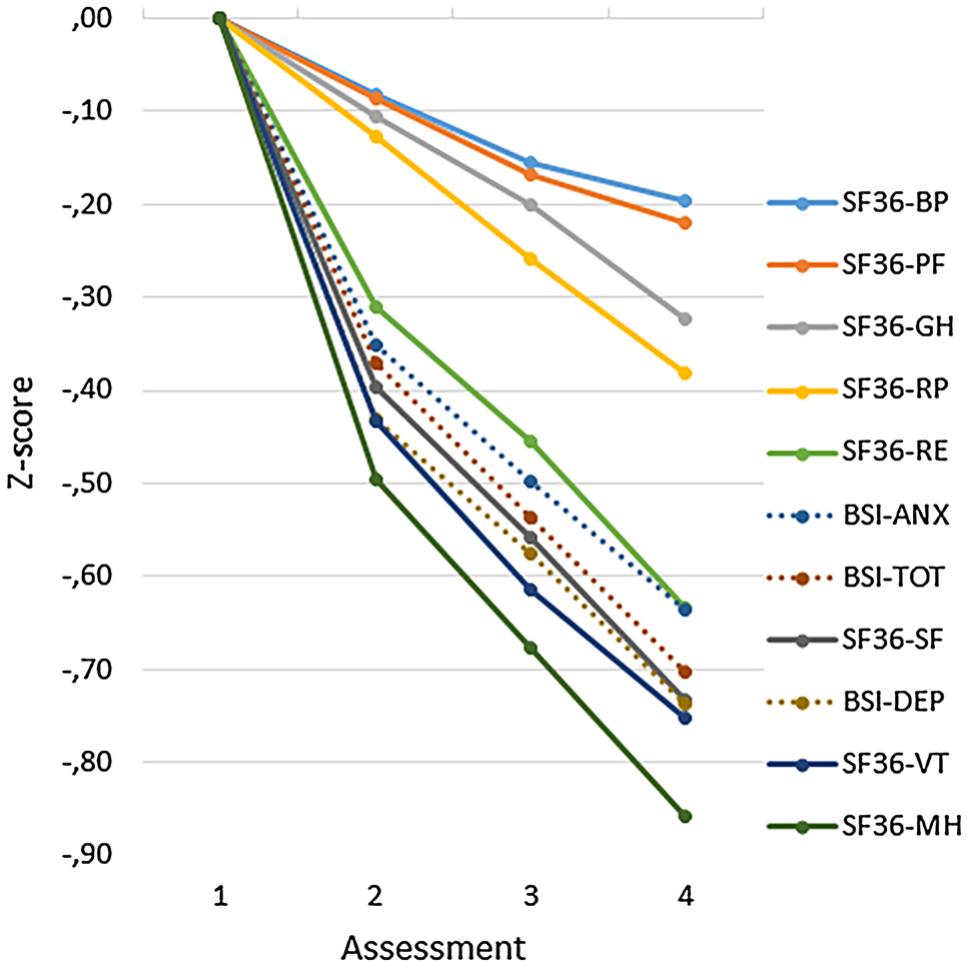
Course over time of standardized *Z*-scores on the BSI–TOT (total score), two BSI subscale scores (DEP and ANX) and eight SF-36 scales for the group with four assessments (*n* = 1453); the order in the legend corresponds to the ranking at the last assessment

A similar analysis of BSI total score and all subscales of the SF-36 revealed a main effect for time [*F*(3, 1460) = 283.42; *p* < .001, *η*^2^ = .368], a main instrument effect [*F*(8, 1455) = 33.36; *p* < .001, *η*^2^ = .155], and a significant interaction effect for instrument-by-time [*F*(24, 1439) = 26.59; *p* < .001, *η*^2^ = .307]. Pairwise comparison of the BSI total score with SF-36 scores reveals that the decrease in score on the BSI differs from all the SF-36 scales, except for the RE-scale (Role limitations due to Emotional problems). Figure 2 illustrates the pattern of change in BSI and SF-36 scale scores over time. It appears that two groups of SF-36 scales can be distinguished regarding how much change they detect. The scales primarily associated with the mental component (VT, SF, RE, and MH) show more change than the scales primarily associated with the physical component (PF, RP, BP, and GH). Figure 2 also shows change in depression and anxiety according to the BSI depression and BSI anxiety subscales, which is comparable to the BSI total score. Changes on the BSI scales are similar to the mental component scales. Furthermore, we inspected the scores of the samples with three (*n* = 2946) or two assessments (*n* = 5786) and found profiles of scores very similar to Figs. 1 and 2 for these larger samples. All in all, according to these analyses the hypothesized dyssynchrony is absent, the instrument-by-time interaction is merely due to less change on the scales measuring physical health.

**Fig. 3:**
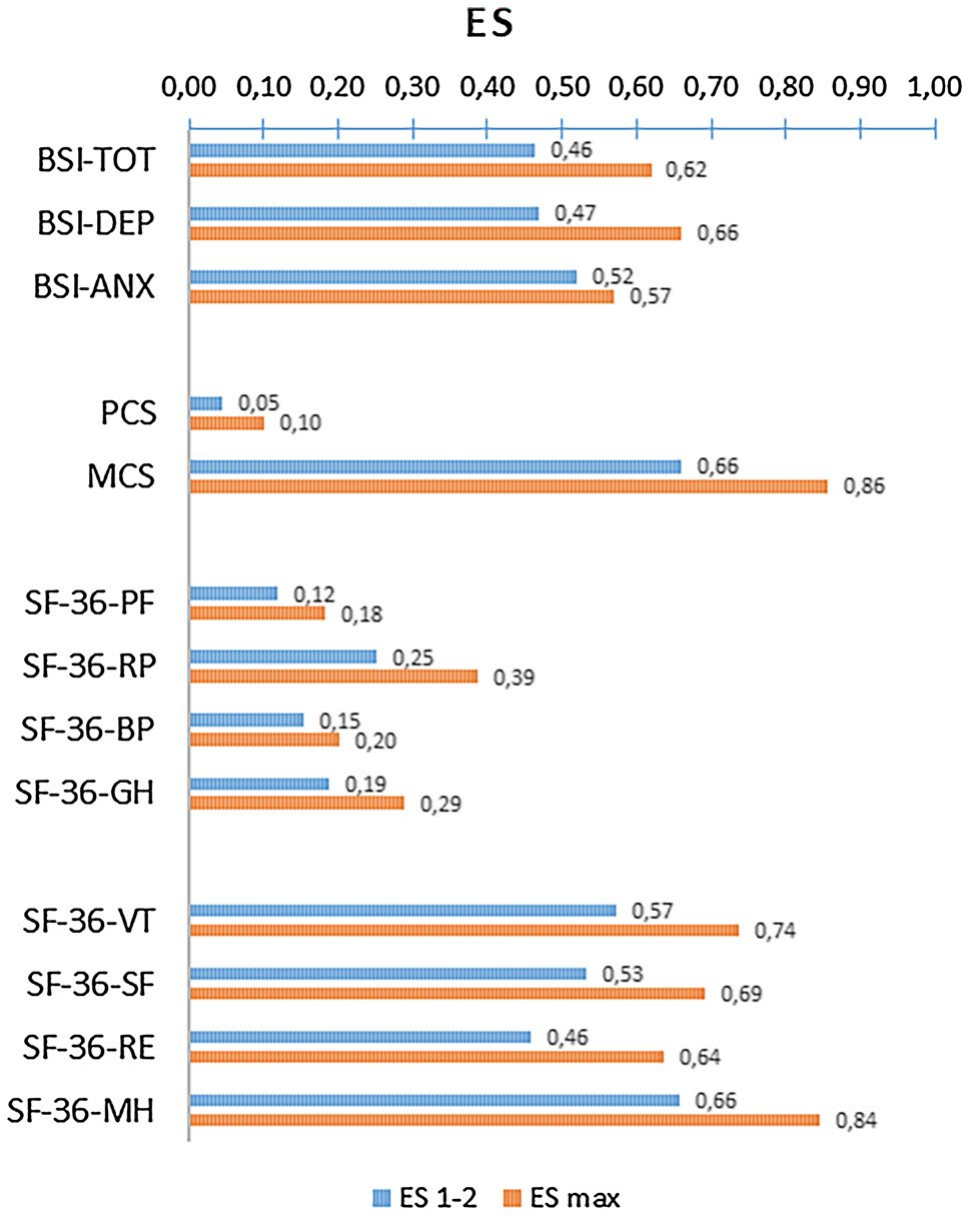
Effect size (ES) of change in the first assessment interval (ES_1–2_) and the maximum change (ES_max_) according to the BSI–TOT (total score), two BSI subscales (DEP and ANX), the SF-36 Physical (PCS) and Mental Component Scores (MCS), and the eight SF-36 scales; the order in the legend corresponds to the ranking at the last assessment

Finally, Fig. 3 presents effect sizes (ES) for the difference in BSI total score and depression and anxiety subscales, the SF-36 scales, and component scores over the first measurement interval (ES_1–2_) and the maximum interval from baseline to final assessment of the entire group (ES_MAX_). Again, the scales BP, PF, GH, and RP show the least change (ES_MAX_ = 0.18 to 0.38) and the scales MH, RE, SF, and VT show the most change (ES_MAX_ = 0.64 to 0.84); the change on the BSI total score (ES_MAX_ = 0.62) is closer to the latter group of scales than to the former. Change on the BSI is similar to scales of the SF-36 that demonstrate the largest change and the BSI depression and anxiety subscales show a somewhat larger change than the BSI–TOT.

## Discussion

The main findings of this study are as follows. There is a high correlation between the BSI total score and component and scale scores of the SF-36, especially for the MH scale. This is in spite of the fact that the BSI and the SF-36 use different temporal instructions for the rated time frame: 1 week vs. 4 weeks, respectively. The substantial concordance between the BSI and the MH scale of the SF-36 is not surprising, given the content of the relevant SF-36 items (“Did you feel nervous?,” “Did you feel downhearted and blue?”), which are very similar to BSI items (“Nervousness or shakiness inside,” “Feeling blue”). The MH scale of the SF-36 demonstrated somewhat larger changes than the BSI total scale. A likely explanation for this finding is that the BSI total scale contains a substantial number of items with low relevancy for patients with mood or anxiety disorders (e.g., “The idea that you should be punished for your sins,” “Feeling that you are watched or talked about by others”). Apparently, for common mental disorders, the generic applicability of the BSI is offset by a somewhat diminished ability to demonstrate change.

The hypothesis of a delayed response on the SF-36 was not supported by scores on the BSI and the SF-36. The data showed a similar linear pattern of change over time for the BSI total score and the mental component of the SF-36. We found a diminished response on the physical component score, but not a delayed response. Generally, scale scores of patients changed over time similarly, but to a lesser extent on physical component scales as compared to mental component scales and BSI scales. These results could have been affected by selective loss to follow-up, as four assessments were only available for 25% of the sample with at least two assessments. We compared the pretest scores of the sample with two assessments and the sample with four assessments and these were very similar. Furthermore, we inspected the course of scores over time of the samples with three (*n* = 2946) or two assessments (*n* = 5786) and found for these larger samples profiles that were very similar to Figs. 1 and 2. These results do not suggest that selective data loss explains the findings of the study.

When comparing the BSI with the SF-36 component scores, the physical component score demonstrated little change in this patient sample, and the BSI and the mental component score showed more or less equal amounts of change. This pattern of scores on the SF-36 is of course specific to patients treated for mental health problems. Likely, among patients treated for somatic diseases, the biggest change would occur on other SF-36 scales. For instance, Garratt et al. [44] compared change in score on the SF-36 scales over time for four groups of patients with somatic diseases (low back pain, menorrhagia, suspected peptic ulcer, and varicose veins). They found that the BP and RP scales revealed the largest changes. Likewise, ten Klooster et al. [45] demonstrated in patients with Rheumatoid Arthritis that the BP scale and the PCS showed the largest change in a retest period of six months. Finally, Frendl and Ware [46] reported on a meta-analysis of 185 drug trials in which they examined change on the component scores for fourteen different somatic conditions, and the PCS score showed overall slightly more change than the MCS. The PCS showed the largest change with psoriatic arthritis and rheumatoid arthritis; the MCS showed larger changes in depression and psoriasis. Nevertheless, when treating psychiatric patients, the physical component score of the SF-36 is still informative, as somatic symptoms are important in their own right and can be an important cause for psychological distress [47, 48].

Further research aimed at broadening the scope of treatment outcome in mental health care research is needed and should focus on other concepts with potential relevance, such as the recovery concept [49]. Alternatively, in the direction of greater specificity, disorder-specific measurement instruments may yield more precise information on treatment gains [50]. The present findings of greater changes on the BSI depression and BSI anxiety subscales—in spite of their brevity—lend support to this suggestion. Finally, the more recent development of item response theory-based computer adaptive tests (CAT), such as the PROMIS assessment battery [51], may prove fruitful for outcomes research, as it allows for a more efficient assessment, without diminishing reliability which is usually associated with brief questionnaires.

A strength of the present study is its use of longitudinal data collected in everyday clinical practice, which enhances the generalizability of the findings. The size of the dataset implies ample statistical power to find differences between the outcome domains. On the other hand, the use of data collected under real-life circumstances yields less experimenter control, resulting in varied assessment intervals and substantial loss of data over time. Consequently, it is likely that treatment outcomes in the present study are somewhat inflated by selective loss of retest data, as patients who finished treatment unsuccessfully may decline to be reassessed. However, for a head-to-head comparison of outcome measures, the present data are very suitable, especially as the availability of lengthy assessments trajectories—four repeated assessments for a substantial number of patients—allowed for the investigation of synchrony of change on outcome domains.

Regarding the concordance between the BSI and the SF-36, it should be noted that the correlation coefficients presented in Table 3 may be a conservative estimate of the actual concordance of the underlying concepts of the instruments. The reliability of the BSI and the SF-36 scales determines the upper limit of their correlation according to the formula ${r}_{\mathrm{max}}= \sqrt{{r}_{xx}{r}_{yy}}$ [52]. The correlation between two scales can be corrected for their unreliability [53] with the formula ${r}^{*}=\frac{{r}_{xy}}{\sqrt{{r}_{xx}{r}_{yy}}}$ (${r}^{*}$ is the attenuated correlation, ${r}_{xy}$ the correlation between the scales and ${r}_{xx}$ and ${r}_{yy}$ are the test–retest reliability coefficients of the scales). With *r* = .82 for the Dutch version of the BSI–TOT score [13] and *r* = .80 for the SF-36-MCS [54], the correlation between the BSI and the MCS would increase from *r* = .61 to ${r}^{*}$ =.75 and for EWB from *r* = .75 to ${r}^{*}$ = .93, indicating that the measured concepts are even more concordant than the unattenuated correlation coefficients of Table 3 reveal.

Finally, the present study focused on whether both instruments assessed change of similar size and pace. While the change appears to be of similar size and had a synchronous course, this head-to-head comparison leaves unclear whether highly similar (latent) variable(s) or dimensions were assessed. In line with this, further research is needed to reveal for which population groups and in which situations one instrument is more advantageous compared to another [55].

## Conclusion

We found correspondence but also significant differences between the BSI and the SF-36: change according to the BSI was similar to the mental component score (and its scales) of SF-36, but patients changed less on the physical component score and scale when compared to the mental component scores. Generally, the BSI and the SF-36 demonstrated a comparable degree of change in groups of patients, and this change occurs in similar size and pace. However, the profile of scores yielded by the SF-36 offers a more complete and more detailed clinical picture of the problems of individual patients, due to the additional domain of physical health.

Thus, the findings illustrate that there is considerable overlap between what is measured with the BSI and the SF-36, but also that each instrument contributes specific information regarding benefits from treatment. The BSI and the mental health component of the SF-36 offer similar specific information on symptom reduction or mental health gains. But the SF-36 clearly measures a broader construct and change on the physical component (and its scales) diverges from change on the SF-36 mental component as well as from change on the BSI. Finally, if the current findings regarding the substantial correlation between the mental component score and the BSI would be replicated with patients who are treated for somatic problems, the mental component scores of the SF-36 could be used to capture concurrent changes in psychological health.
